# 7-Hydroxymitragynine and Nicotine Pouch Withdrawal Syndrome: A Case Report

**DOI:** 10.7759/cureus.98386

**Published:** 2025-12-03

**Authors:** Akshay Sharma, Beena S Nair, Sudhakar Pemminati

**Affiliations:** 1 Department of Biomedical Education, California Health Sciences University College of Osteopathic Medicine, Clovis, USA; 2 Department of Psychiatry, University of California San Francisco, Fresno, USA

**Keywords:** 7-hydroxymitragynine, kratom addiction, nicotine withdrawal, psycho-behavioral, psychodiagnostics and therapy

## Abstract

Novel psychoactive substances (NPSs) such as kratom and oral nicotine pouches present new challenges in clinical management, particularly regarding dependence and withdrawal. We report a case of severe withdrawal from concentrated 7-hydroxymitragynine (7-OH), a potent kratom alkaloid, and high-dose oral nicotine pouches, following long-term use in a patient with a history of polysubstance use. The patient developed acute, overlapping opioid-like (insomnia, myalgia, anxiety, diaphoresis, and gastrointestinal upset) and nicotinic (intense craving, irritability, restlessness, and difficulty concentrating) withdrawal symptoms. These symptoms rapidly escalated to severe agitation, psychosis, and respiratory compromise requiring intubation and intensive care. The clinical course was further complicated by failed outpatient management, precipitated withdrawal after initiation of buprenorphine, and eventual discharge against medical advice (AMA). This case report highlights the diagnostic and therapeutic complexities of managing withdrawal from novel psychoactive substances, the need for multidisciplinary and flexible approaches, and the importance of systematic assessment and harm reduction as these products become increasingly prevalent.

## Introduction

The global rise in novel psychoactive substance (NPS) use has introduced new challenges for clinicians in recognizing and managing withdrawal syndromes, a trend reflected in international surveillance reports such as the United Nations Office on Drugs and Crime (UNODC) World Drug Report 2024 [1]. Among these, kratom (*Mitragyna speciosa*) is a tropical tree native to Southeast Asia, where its leaves have been used for centuries for their stimulant and analgesic properties, as well as for the management of pain and opioid withdrawal symptoms [2-6]. The plant contains over 40 alkaloids (mitragynine, 7-hydroxymitragynine (7-OH), speciogynine, paynantheine, and speciociliatine), with 60%-70% of total alkaloid content being mitragynine and 7-hydroxymitragynine. These alkaloids act as partial agonists at μ-opioid receptors and also modulate adrenergic, serotonergic, and dopaminergic pathways, resulting in dose-dependent stimulant and opioid-like effects [2,3,6].

In recent decades, kratom has gained popularity and unregulated availability in Western countries, where it is marketed as a dietary supplement and used for self-management of chronic pain, opioid withdrawal, and mood disorders [2,4,5,7-10]. Its legal status remains controversial, with regulatory agencies such as the US Drug Enforcement Administration and the US Food and Drug Administration (FDA) considering it a drug of concern due to its abuse potential and lack of standardized safety data [2,4,11-13].

Kratom use has been associated with a range of adverse effects, including physical and psychological dependence, withdrawal symptoms, seizures, and organ toxicity [2,5,7,9,10,14,15]. Of particular concern is the emerging recognition of kratom-induced liver injury, which is typically cholestatic or mixed in pattern and may present with jaundice, pruritus, and abdominal discomfort [2,5,14]. Case reports and cohort studies have shown that symptom onset can occur 2-49 days after exposure, often requiring hospitalization, with histopathology frequently revealing portal inflammation and bile duct injury that may mimic autoimmune cholangiopathies [14].

Kratom withdrawal symptoms reflect the predominant effects at higher doses and may include myalgias, anxiety, insomnia, gastrointestinal distress, irritability, and restlessness. Understanding these dose-dependent effects is essential for accurate diagnosis and management of kratom withdrawal.

Dose-dependent symptoms may include the following: stimulant-like effects, including increased alertness, sociability, and energy, at low doses (1-5 g) and opioid-like effects, such as sedation, euphoria, analgesia, and, in some cases, respiratory depression, at high doses (5-15 g).

An important distinction exists between plant-derived kratom and concentrated 7-hydroxymitragynine (7-OH) extracts. Natural kratom contains relatively low levels of 7-OH, whereas commercial extracts may contain enriched or semi-synthetic 7-OH, resulting in substantially greater μ-opioid receptor potency, higher dependence risk, and more severe withdrawal presentations [2,7,10,14]. This difference in alkaloid concentration helps explain the variability and severity of cases described in Western clinical settings.

Further research is needed to clarify the mechanisms, dose-response relationships, and long-term safety of kratom use [2,4,5,7,9,10,14]. Oral nicotine pouches, marketed as “tobacco-free” smokeless nicotine products, deliver nicotine through oral mucosal absorption, typically containing 2-12 mg of nicotine per pouch [15]. Despite claims of reduced harm compared to traditional tobacco, these products contain nicotine concentrations sufficient to induce dependence and clinically significant withdrawal [15]. Withdrawal symptoms mirror those seen in cigarette cessation, including cravings, irritability, restlessness, dysphoria, and difficulty concentrating [16].

The coexistence of kratom and nicotine pouch use introduces unique diagnostic and therapeutic challenges, particularly in acute withdrawal presentations [2,3,6,15,17]. Both substances act on distinct but interconnected neurobiological pathways: opioid receptor activity modulates dopaminergic tone in the mesolimbic system, while nicotine exerts dopaminergic effects through nicotinic acetylcholine receptors [2,3,6,17]. The convergence of these pathways can amplify reward signaling, reinforcing polysubstance use and complicating withdrawal syndromes [2,3,6,17].

Despite rising prevalence, there remains a paucity of controlled studies addressing clinical management of withdrawal from kratom and nicotine pouches, as no standardized treatment protocols currently exist [7,8,15]. Case reports remain the primary source of clinical guidance [7,8,15]. Here, the presented case is that of concurrent withdrawal from 7-hydroxymitragynine and nicotine pouches in a polysubstance user, and the current literature is reviewed to contextualize management considerations and harm reduction strategies [2,4,5,7,9-15].

## Case presentation

A 39-year-old man with a history of polysubstance use disorder presents for management of kratom and nicotine withdrawal. His substance use history includes alcohol (first drink at age 24, drink of choice unknown, now sober for two years following a driving under the influence (DUI) arrest, denies current use), tobacco (using four nicotine pouches daily for approximately the past year), kratom (liquid form, first used at age 36, daily use until age 39, denies current use), and 7-hydroxymitragynine (oral, 700-800 mg/day, initiated at age 39, daily use including the last 30 days, last use on 7 am on morning of presentation to the emergency department (ED)). His medical history was notable for prediabetes (diagnosed on 5/26/2015, no active treatment), asthma (diagnosed on 5/6/2008, no active treatment), childhood attention deficit hyperactivity disorder (ADHD), combined presentation (diagnosed on 8/30/2016, no active treatment), and a history of musculoskeletal pain (prescribed medications include meloxicam 7.5 mg oral tablet and tizanidine 2 mg oral tablet, which the patient reported that he was not taking on 9/29/2022). He denies any family history of substance use disorder or mental health conditions. The patient reported living with his wife and daughter. He worked as a foreman in an electrical company for 12 years; his highest education was grade school. He denies any history of trauma (physical, sexual, or domestic violence) and no legal involvement or active legal mandates. He reported no history of psychiatric conditions or drug/alcohol use disorders in his family. He presented to the ED from a rehab for management of kratom and nicotine withdrawal. At presentation, he reported stable housing and employment, moderate family support, and a spouse who was aware of and supportive of his recovery. At the bedside, the patient’s wife stated that he was excited to go to the rehab facility to get help.

The patient began using kratom (unknown dosage, daily use) three years prior for unspecified musculoskeletal pains caused by work-related strain after hearing about it on a podcast. About eight months before the presentation, he was introduced to concentrated 7-OH extracts, which he found highly effective for pain, but quickly developed physical dependence. Over the past month, he escalated to 10 oral tablets of 80 mg 7-OH daily (approximately 800 mg/day). He had a previous history of smoking (unspecified) with no recent smoking reported. As of approximately the past year, he reported daily use of up to four tobacco-free nicotine pouches, with each containing 6 mg of nicotine (approximately 24 mg/day).

He self-referred to chemical dependency (CD) services, stating, “I became dependent on 7-OH … They sell it everywhere … I’m spending $150 a day … My body starts shaking as soon as it starts to wear off … After doing some research, I found out that it is a synthetic opioid … I tried quitting on my own, but nothing has worked … I need professional help.”

He identified his goal as complete abstinence. He described previous withdrawal symptoms as “difficulty breathing, tickling sensation in bones, restlessness, anxiety, insomnia, and appetite changes,” consistent with mixed opioid-nicotinic withdrawal features. He acknowledged the negative impact of 7-OH, stating, “I don’t have a problem with drugs and thought this substance was safe because it is accessible over the counter … I was wrong … Had I known the risk and dangers of this substance, I would have never tried it….”

He started using kratom for back pain and, eight months prior to presentation, switched to 7-OH for ease of consumption, using 10 tablets of 80 mg (800 mg/day). Prior to arrival, he attempted to taper, using 600 mg the day before and 300 mg the morning of presentation, with the last use at 07:00. He denied other illicit substance use.

He had previously attempted to quit independently, but withdrawal symptoms became intolerable within 6-8 hours. He saw a physician who prescribed buprenorphine-naloxone (8-2 mg SL film, one film sublingually twice daily as needed), but after taking it one hour after 7-OH, he experienced precipitated withdrawal and severe anxiety, which deterred further attempts, as buprenorphine’s high receptor affinity displaces full agonists (like 7-OH), precipitating withdrawal if administered too soon [2,3,11,12]. Outpatient medications included naloxone 4 mg/actuation nasal spray, which was not used, and the patient continued to use 7-OH. Vivitrol was recommended after 14 days of opioid-free, with inpatient detoxification advised due to his prior experience with suboxone and concerns about managing withdrawal as an outpatient. Screening tools revealed a Patient Health Questionnaire‐2 (PHQ‐2) score of 0 and a PHQ-9 score of 13, indicating moderate depressive symptoms [18,19].

Interventions and management

The patient was transferred from an inpatient rehabilitation facility on the same day he was admitted to the emergency department (ED) due to acute withdrawal symptoms following the abrupt cessation of concentrated 7-OH kratom extracts. He reported last using 600 mg the day prior and 300 mg the morning of presentation (last dose at 07:00), in an attempt to self-taper. He also reported daily use of up to four tobacco-free nicotine pouches, each containing 6 mg of nicotine.

On arrival, he was alert and oriented but appeared visibly restless and ill, with pupils reactive to light and no significant miosis. Vital signs showed mild hypertension (blood pressure (BP): 144/62 mmHg) and tachycardia (heart rate (HR): 127 bpm). Mental status examination revealed mild psychomotor agitation, restlessness, and irritability; he denied hallucinations, seizures, and suicidal or homicidal ideation and was fully oriented to person, place, time, and situation. Laboratory studies were unremarkable (Table 1 and Table 2). Urine toxicology was negative for opioids, benzodiazepines, and stimulants, supporting the clinical suspicion of withdrawal from 7-OH and nicotine rather than other substances (Table 3 and Table 4).

**Table 1:** Hematology and differential laboratory values on arrival Hgb: hemoglobin

Test	Result	Reference range	Interpretation
Hgb	14 g/dL	13-17 g/dL	Normal
Hematocrit	39%	39%-51%	Lower end of normal
White blood cell count	9.7 K/uL	3.7-11.1 K/uL	Normal
Red blood cell count	4.39 M/uL	4.10-5.70 M/uL	Normal
Mean corpuscular volume	89 fL	80-100 fL	Normal
Red cell distribution width and red blood cell	11.9%	11.5-16.5%	Normal
Platelet count	342 K/uL	140-400 K/uL	Normal
Nucleated red blood cells	≤0/100 WC	≤0/100 WC	Normal
Neutrophils, automated count	6.7 K/uL	1.8-7.9 K/uL	Normal
Lymphocytes, automated count	2.3 K/uL	0.9-3.2 K/uL	Normal
Monocytes, automated count	0.7 K/uL	0.3-0.9 K/uL	Normal
Eosinophils, automated count	0.1 K/uL	0.0-0.4 K/uL	Normal
Basophils, automated count	0 K/uL	0.0-0.1 K/uL	Normal

**Table 2:** Basic metabolic panel upon admission (Chem 7)

Test	Result	Reference range	Interpretation
Estimated glomerular filtration rate	>60 mL/min/1.73 m²	≥60 mL/min/1.73 m²	Normal
Sodium	138 mEq/L	135-145 mEq/L	Normal
Potassium	3.5 mEq/L	3.5-5.3 mEq/L	Lower end of normal
Chloride	103 mEq/L	100-111 mEq/L	Normal
Bicarbonate	25 mEq/L	24-33 mEq/L	Normal
Anion gap, serum/plasma	10 mEq/L	5-16 mEq/L	Normal

**Table 3:** General chemistry and toxicology screen results upon admission

Test name	Result	Reference range	Interpretation
Blood urea nitrogen	9 mg/dL	7-27 mg/dL	Normal
Glucose, random	141 mg/dL	60-159 mg/dL	Normal
Creatinine	0.65 mg/dL	≤1.34 mg/dL	Normal
Troponin I, high sensitivity	<2 ng/L	≤20 ng/L	Normal
Aspartate aminotransferase	16 U/L	10-40 U/L	Normal
Alanine aminotransferase	17 U/L	10-47 U/L	Normal
Bilirubin, total	0.4 mg/dL	0.2-1.2 mg/dL	Normal
Alkaline phosphatase	69 U/L	37-117 U/L	Normal
Lipase	19 U/L	10-140 U/L	Normal
Calcium	9.8 mg/dL	8.8-10.5 mg/dL	Normal
Magnesium	1.8 mg/dL	1.7-2.3 mg/dL	Normal
Salicylate level	<3 mg/dL	≤30 mg/dL	Normal
Acetaminophen level	<3 ug/mL	0-30 ug/mL	Normal
Ethyl alcohol level, blood	<10	mg/dL	Normal
Ammonia, blood	40 umol/L	16-53 umol/L	Normal
Creatine kinase	155 U/L	≤200 U/L	Normal

**Table 4:** Urine toxicology screen results THC: tetrahydrocannabinol

Test	Result	Reference range	Interpretation
Amphetamines, urine, screening	Negative	Negative	Negative
Methamphetamine, urine, qualitative	Negative	Negative	Negative
Barbiturates, urine, qualitative	Negative	Negative	Negative
Benzodiazepines, urine screen	Negative	Negative	Negative
Cocaine, urine, qualitative	Negative	Negative	Negative
Methadone, urine screen	Negative	Negative	Negative
Opiates, urine, screening	Negative	Negative	Negative
Oxycodone, urine, qualitative, screening	Negative	Negative	Negative
Phencyclidine, urine screen	Negative	Negative	Negative
THC, urine, screening	Negative	Negative	Negative
Fentanyl metabolite screen, urine	Negative	Negative	Negative

During the course of emergency department observation, the patient exhibited escalating signs of anxiety and restlessness, characterized by frequent movement, verbal expressions of discomfort, and difficulty remaining seated. These behaviors prompted further evaluation for potential physiological or psychological triggers. Poison control was consulted, which further supported the diagnosis of withdrawal and recommended starting buprenorphine 4 mg cautiously and benzodiazepines (diazepam injectable 5 mg given twice and midazolam (preservative-free (PF)) injectable 4 mg). The patient became more diaphoretic, restless, agitated, aggressive, and combative, with disorientation and possible hallucinations (calling for people who were not present). He was given diazepam injectable 10 mg once for agitation, but his condition did not improve, and he eventually required four-point restraints. Due to further decompensation and inability to protect his airway, the patient was intubated using ketamine and rocuronium for airway control.

The administered dosages were as follows: ketamine was given in two doses, 136 mg and 45 mg (totaling 181 mg), and rocuronium was administered as a single dose of 110 mg; the doses were determined based on his body weight. Ketamine was provided as an induction agent, and rocuronium was used as the paralytic. No additional doses or repeat administrations were documented. Repeat vitals showed hypertension, and chest X-ray confirmed appropriate placement of the endotracheal tube; an orogastric tube was also placed. The bed head was elevated, and sedation and pain management were initiated as standard supportive measures. Dexmedetomidine in normal saline 400 mcg/100 mL (4 mcg/mL) IV (continuous) was ordered to supplement propofol 1000 mg/100 mL IV (continuous), and a midazolam (PF) in NS 1 mg/mL IV premix was started for sedation. The patient was admitted to the intensive care unit (ICU) in critical condition.

Toxicology consultation provided education on kratom and included a differential diagnosis of opioid withdrawal and alcohol withdrawal/delirium tremens. Alcohol withdrawal was ruled out based on a negative ethanol screen and history. Recommendations included continued supportive care and consideration of phenobarbital 10 mg/kg IV (patient was given a single loading dose).

Sedation was managed and weaned, with the patient initially at Glasgow Coma Scale (GCS) 3T. Tube feeds were started. The patient remained very agitated, requiring midazolam 8 mg, followed by continuous infusions of propofol 1000 mg/100 mL IV (continuous), dexmedetomidine in normal saline 400 mcg/100 mL (4 mcg/mL) IV premix (continuous), and fentanyl in normal saline 10 mcg/mL IV (continuous). The next day, oxycodone 10 mg, diazepam 10 mg, and dexmedetomidine in normal saline 400 mcg/100 mL (4 mcg/mL) IV (continuous) were administered in an attempt to wean sedation and facilitate extubation. The patient passed a spontaneous breathing trial with a GCS of 11.

With toxicology consultation, his home medication for mood stability while undergoing substance use disorder treatment, quetiapine 50 mg, twice daily, was restarted to assist with agitation and any underlying behavioral health issues. Sedatives were weaned, with diazepam tapered first with 5 mg q12 for two doses and a plan to be followed by opioids. The patient was downgraded to the telemetry floor.

The patient developed fevers; urinalysis was negative for infection, and blood cultures were drawn. Acetaminophen (1000 mg IV and PO q8 hours) and cooling measures were provided. Fever was most likely due to aspiration pneumonitis versus pneumonia related to intoxication, intubation, and mechanical ventilation. Ceftriaxone 2 g once daily and metronidazole 500 mg every eight hours were started empirically, with a good clinical response.

Quetiapine was increased to 100 mg PO QHS in addition to 50 mg PO QAM. The patient began to demonstrate confusion and attempted to pull out lines. Alternatives, including haloperidol 5 mg, diazepam 5 mg, lorazepam 2 mg, and verbal redirection, were attempted, but restraints had to be re-ordered for patient safety. He exhibited agitation with line-pulling behavior, necessitating re-initiation of restraints. Psychiatry was consulted, and the morning quetiapine was discontinued, with 50 mg twice daily, as needed for breakthrough agitation, and 100 mg PO QHS continued. Lorazepam 0.5 mg PO BID was prescribed for anxiety and restlessness. Nicotine replacement was considered (but not prescribed) due to his prior use of four pouches of tobacco-free nicotine pouches per day (6 mg nicotine per pouch, totaling 24 mg/day) for approximately the past year.

Oxycodone was continued to be weaned with a plan to start buprenorphine (4 mg)-naloxone (1 mg) PO BID after 12 hours of oxycodone, with plans to increase withdrawal as tolerated. The patient agreed to try suboxone while in the hospital. On further discussion the following day, the patient was calm and cooperative, drowsy but oriented ×4. He denied use of alcohol and reported abstinence from 7-OH and tobacco-free nicotine pouches. On the mental status examination, he denied depression, anxiety, or any suicidal/homicidal ideation or auditory/visual hallucinations.

The patient was counseled on symptom management and was offered options of pharmacologic support, including clonidine for autonomic symptoms, hydroxyzine for anxiety, and nicotine replacement therapy for nicotine withdrawal. Behavioral strategies, hydration, and education about the expected course of withdrawal were also discussed. Despite these interventions, he expressed a strong preference to return to his prior routine and declined further hospital admission, leaving against medical advice (AMA) after several hours of observation.

At the time of discharge, the patient was prescribed a comprehensive regimen to address opioid withdrawal, relapse prevention, and associated symptoms. The discharge medications included buprenorphine 2 mg standard release sublingual tablet, directed for a 14-day taper; buprenorphine 8 mg standard release sublingual tablet, directed for a 14-day taper; buprenorphine-naloxone 8-2 mg sublingual film, to be applied sublingually twice daily as needed; clonidine 0.1 mg oral tablet, prescribed with a scheduled taper (one tablet orally four times daily for four days, then three times daily for three days, then twice daily for three days, and finally once daily in the morning for four days); naloxone 4 mg/actuation nasal spray, provided for emergency opioid overdose reversal; quetiapine 100 mg oral tablet, once nightly as needed (renewal requested); and quetiapine 50 mg tablet (renewal requested; frequency not specified).

The inclusion of naloxone nasal spray aligns with harm reduction recommendations for patients at risk of opioid overdose, and the use of clonidine addresses autonomic symptoms during withdrawal. Quetiapine was continued as needed for sleep or anxiety, recognizing the need for ongoing management of co-occurring symptoms.

Outpatient follow-up and harm reduction counseling were recommended, including engagement with addiction medicine, psychiatry, and community support resources. The patient was advised to monitor for escalating withdrawal symptoms, maintain hydration and nutrition, and return promptly if symptoms worsened or new psychiatric or medical concerns arose.

At outpatient follow-up with addiction medicine and recovery services five days after discharge, the patient reported ongoing engagement in individual services and maintained abstinence from opioids since the morning of hospital admission. He denied experiencing any cravings or urges to use opioids and expressed feeling stable, with no current concerns about relapse. The patient had not been regularly attending community recovery support meetings but had otherwise followed the recommended treatment plan.

During the visit, the patient raised concerns about poor sleep quality, reporting difficulty falling and staying asleep, and averaging approximately one hour of sleep per night over the past week. He inquired about alternative medication options for sleep. Brief education was provided on sleep hygiene and the impact of substance use disorder on sleep patterns. The patient was reminded of his upcoming medical appointment to address medication concerns further.

Additionally, the patient expressed interest in an outpatient group programs and was recommended to consider a day treatment level of care. He requested additional time to consider his options and agreed to contact the clinic once he had decided on participation. Ongoing monitoring and support were arranged to address both substance use and co-occurring symptoms, in line with best practice guidelines for addiction medicine. The substances used in this case are summarized in Table 5. The timeline for the medications and interventions, including dosages and frequencies, is provided in Table 6.

**Table 5:** Summary of key substances in this case DEA: Drug Enforcement Administration, FDA: Food and Drug Administration, 7-OH: 7-hydroxymitragynine

Substance	Active compound(s)	Mechanism of action	Clinical effects	Risks/adverse events	Legal/regulatory status
Kratom (Mitragyna speciosa) [2,3,9,10,15]	Mitragynine, 7-OH (minor alkaloid)	Partial agonist at μ-opioid receptors; adrenergic and serotonergic activity	Euphoria, analgesia, stimulation (low dose), sedation (high dose)	Dependence, withdrawal (opioid-like), hepatotoxicity, seizures, arrhythmias	Legal at the federal level but restricted in several states; listed as a drug of concern by the DEA
7-OH [7,8,14,15,17]	Potent opioid alkaloid (metabolite of mitragynine)	Potent μ-opioid receptor agonist (preclinical data suggest higher potency than morphine)	Strong analgesia, sedation, euphoria	High abuse potential, dependence, opioid-like withdrawal, and respiratory depression	7-OH is a metabolite of mitragynine, not typically sold as a stand-alone FDA-recognized compound; commercial extracts may contain enriched or semi-synthetic 7-OH, which is not FDA-approved; research chemical
Nicotine pouches [16]	Nicotine (synthetic or extracted)	Nicotinic acetylcholine receptor agonist (↑dopamine release)	Stimulation, increased alertness, relaxation, and appetite suppression	Nicotine dependence, cardiovascular risks, hypertension, and oral irritation	Legal; regulated as a tobacco product by FDA regulations under the Center for Tobacco Products

**Table 6:** Timeline of events, medications, and interventions ED: emergency department, GCS: Glasgow Coma Scale, PRN: as needed, QHS: every night at bedtime, SL: sublingual, PO: oral route, BID: two times a day, ICU: intensive care unit, IV: intravenously, SBT: spontaneous breathing trial, 7-OH: 7-hydroxymitragynine, AMA: against medical advice

Date	Clinical events and status	Medications and interventions
8/08	Clinical events:	Discrete doses:
	ED arrival with acute 7-OH + nicotine withdrawal; restless, tachycardic	Diazepam 5 mg IV (×2)
	Escalating agitation followed by hallucinations	Midazolam 4 mg IV
	4-point restraints applied	Buprenorphine 4 mg attempted (per tox)
	Intubated for airway protection	Diazepam 10 mg IV for severe agitation
		Ketamine 136 mg IV
		Ketamine 45 mg IV
		Rocuronium 110 mg IV (paralytic)
		Continuous infusions:
		Propofol infusion 1000 mg/100 mL
		Dexmedetomidine infusion 4 mcg/mL
		Midazolam infusion 1 mg/mL
8/09	Clinical status:	Discrete doses:
	ICU course; sedated, GCS 3T	Phenobarbital 10 mg/kg IV (single dose)
	Tube feeds started	Oxycodone 10 mg PO
		Diazepam 10 mg PO
		Continuous infusions:
		Propofol infusion (continued)
		Dexmedetomidine infusion (continued)
		Fentanyl infusion 10 mcg/mL (started)
8/10	Clinical events:	Discrete doses:
	Attempts at sedation wear off; agitation	Quetiapine 50 mg BID restarted (home medication)
	Passed SBT; extubated, GCS 11	Quetiapine 100 mg QHS
		Midazolam 8 mg IV PRN
8/11	Clinical events:	Discrete doses:
	Fever workup (likely aspiration)	Acetaminophen 1000 mg IV/PO q8h
	Agitation with line-pulling and resulting restraints	Ceftriaxone 2 g IV daily (empiric)
	Psychiatry consult	Metronidazole 500 mg IV q8h
		Haloperidol 5 mg PO/IV PRN
		Diazepam 5 mg PO PRN reintroduced
		Lorazepam 2 mg IV/PO PRN
		Duration/plan:
		Sedation gradually weaned
8/12	Clinical status:	Discrete doses:
	Improved orientation; calm in the interview	Quetiapine 50 mg BID PRN (AM dose stopped)
	Nicotine replacement is considered	Quetiapine 100 mg QHS continued
	Oxycodone tapers are ongoing	Lorazepam 0.5 mg PO BID
		Plan:
		Buprenorphine 4 mg BID after 12 hours opioid-free (patient agreeable)
8/13	Clinical status:	Prescribed medications:
	Patient clinically stable; denies cravings; reports poor sleep	Clonidine 0.1 mg taper prescribed
	Leaves AMA	Buprenorphine (2 mg and 8 mg SL tablets)
		Buprenorphine-naloxone 8/2 mg SL films
		Naloxone nasal spray 4 mg
		Quetiapine 100 mg QHS PRN
		Quetiapine 50 mg PRN (renewal)
		Follow-up:
		Follow-up addiction medicine arranged

## Discussion

This case highlights the diagnostic and therapeutic challenges associated with withdrawal from novel psychoactive substances, particularly in the context of polysubstance use. Kratom withdrawal closely resembles opioid withdrawal in both neurobiological mechanisms and clinical presentation, as 7-hydroxymitragynine acts as a potent partial μ-opioid receptor agonist [2,3]. Patients may experience symptoms such as myalgias, anxiety, insomnia, and gastrointestinal upset, with severity influenced by dosage, frequency, and concurrent substance use [9,10,15]. In contrast, withdrawal from nicotine pouches is characterized by dopaminergic dysregulation within the mesolimbic pathway, leading to irritability, cravings, restlessness, and difficulty concentrating [16]. When these syndromes overlap, patients may present with a mixed withdrawal picture that challenges diagnostic clarity, especially in a patient with multiple addictions.

The principal alkaloids in kratom, mitragynine and 7-hydroxymitragynine, act as partial μ-opioid receptor agonists and produce opioid-like withdrawal symptoms such as myalgia, insomnia, anxiety, and gastrointestinal distress, along with stimulant-like features including irritability and restlessness [3,8,14]. The intensity of withdrawal is closely linked to the frequency and duration of use [9,10]. Unlike opioid use disorder, there are no FDA-approved pharmacotherapies for kratom withdrawal. Management is primarily symptomatic, utilizing agents such as clonidine, hydroxyzine, and benzodiazepines [7,15]. Although buprenorphine and methadone have shown potential benefit in case reports and preclinical studies, they are not part of established guidelines [7,15,16].

Oral nicotine pouches, marketed as tobacco-free alternatives, deliver sufficient nicotine to induce dependence. Withdrawal symptoms, including irritability, craving, and restlessness, are similar to those seen with traditional tobacco products and e-cigarettes. While these products may support harm reduction in established smokers, their increasing use among nicotine-naïve young adults and polysubstance users raises significant public health concerns, especially given the limited data on long-term effects and abuse potential [16].

From a management perspective, supportive care and symptomatic treatment remain the mainstay of therapy, as evidence-based pharmacologic protocols for kratom withdrawal are limited. Alpha-2 agonists such as clonidine or lofexidine may help control autonomic symptoms and reduce the need for deep sedation. Early initiation of buprenorphine or buprenorphine-naloxone, once adequate withdrawal is documented, may also shorten the overall course and decrease the risk of rebound agitation. Currently, clinical consensus is lacking. Adjuncts such as gabapentin or home antipsychotic regimens can support anxiety, agitation, and insomnia, although their sedating effects require caution. Nicotine replacement therapies, such as lozenges, patches, or gums, and non-nicotine pharmacologic aids, such as varenicline or bupropion, may mitigate withdrawal from oral pouches. Importantly, psychosocial interventions, patient education, and harm reduction strategies play a central role in addressing both acute withdrawal and long-term relapse prevention [5,12]. This case also reinforces the importance of minimizing iatrogenic respiratory compromise through structured sedation weans, objective sedation scoring, and close respiratory monitoring during withdrawal. Early psychiatry involvement remains essential, both for agitation management and to reduce the likelihood of discharge against medical advice (AMA).

## Conclusions

This case highlights the intensity and clinical complexity of severe kratom withdrawal, particularly when agitation, respiratory compromise, and co-occurring psychiatric needs overlap. Successful management requires a multimodal approach that includes structured sedation weaning, timely consideration of buprenorphine, and close collaboration between critical care, toxicology, and psychiatry. The rapid shift from agitation to acute respiratory failure in this patient emphasizes the importance of early airway planning during withdrawal treatment. His AMA discharge further underscores the need for clear communication, anxiety management, and more standardized kratom withdrawal protocols to support patient engagement and safe follow‑up.
